# Supplementary material for: Computed tomography radiomics-based cross-sectional detection of mandibular osteoradionecrosis in head and neck cancer survivors
Source: medRxiv. 2024 Sep 12:2024.09.11.24313485. Preprint. [Version 1] doi: 10.1101/2024.09.11.24313485 (PMC11419222; doi:10.1101/2024.09.11.24313485)
Supplement: Supplement 1 [file media-1.pdf]

Appendix A. Clinical characteristics of the patients involved in the study.

Characteristic	Value (N=150)
**Demographic Characteristics**	
Mean (Range) Age of Patients at the Time of the Contrast-Enhanced CT Study	62.3 (27-82)
Sex	Male = 131 (87.3%),<br>Female: 19 (12.6%)
Race	White or Caucasian: 135 (90%),<br>Black or African American: 6 (4%),<br>Other: 9 (6%)
Smoking Status	Former: 85 (56.6%),<br>Never: 49 (32.6%),<br>Current: 16 (10.6%)
Mean Smoking Pack Years (SD)	23.3 (29.5)
Alcohol Status	Yes: 53 (35.3%)<br>No: 93 (62%)<br>Former: 4 (2%)
Alcohol Levels *	None-Occasional-Moderate: 136 (90.7%)<br>Heavy: 14 (9.3%)
**Disease Chronology & Survivorship**	
RT End-ORN_0 Interval in Months	Mean: 32.6<br>SD: 41.6<br>Range: 0-282
ORN_0-Mandibulectomy Interval in Months (n=69)	Mean: 19.6<br>SD: 20.6<br>Range: 1-90
ORN_0-ORN_1 Interval in Months	Mean: 10<br>Range: 0-71
ORN_0-Last Follow-up Interval in Years	Mean: 4.6<br>SD: 3.1<br>Range: 0-15
Last Follow-up Status	Alive with ORN: 53 (35.3%)<br>No evidence of ORN: 41 (27.3%)

	Deceased: 56 (37.3%)
**ORN Characteristics**	
ORN Grade (Tsai et al. [18])	Grade 1: 16 (10.7%),<br>Grade 2: 23 (15.3%),<br>Grade 3: 36 (24%),<br>Grade 4: 75 (50%)
ORN Laterality to Tumor Side	Ipsilateral: 127 (84.7%),<br>Contralateral: 23 (15.3%)
ORN Treatment Modalities	Mandibulectomy: 69 (46%)<br>Hyperbaric Oxygen: 79 (52.6%)
**RT Characteristics**	
RT Technique	IMRT: 103 (68.7%)<br>VMAT: 11 (7.3%)<br>IMPT: 9 (6%)<br>Conventional 3D Technique: 1 (0.7%)<br>Not Recorded: 26 (17.3%)
Pre-RT Dental Status	No Dental Intervention: 58 (38.7%)<br>Dental Extraction: 44 (29.3)<br>Edentulous: 12 (8%)<br>Not Recorded: 36 (24%)
Mean Radiation Dose (Range)	66.6 (50-72)
Mean Number of Radiation Fractions (Range)	32.3 (25-42)
The Patient had >1 Head and Neck RT Courses Prior to ORN (Yes/No)	No = 128 (85.3%)<br>Yes = 22 (14.7%)
Therapy Sequence	RT Only: 30 (20%)<br>CRT Only: 73 (48.7)<br>Induction CT followed by CRT: 39 (26%)<br>Induction CT followed by RT Only: 8 (5.3%)
**Tumor Characteristics**	
Tumor Site	Base of Tongue: 43 (28.7%),<br>Tonsil: 41 (27.3%),<br>Tongue: 21 (14%),<br>Floor of mouth: 8 (5.3%),

	Oropharynx: 6 (4%),<br>Parotid: 4 (2.7%),<br>Other Head and Neck Sites: 27 (18%)
Tumor Histology	Squamous Cell Carcinoma: 149 (99.3%),<br>Mucoepidermoid Carcinoma: 1 (0.7%)
T-Stage	T0: 1 (0.7%),<br>T1: 26 (17.3%),<br>T2: 44 (29.3%),<br>T3: 29 (19.3%),<br>T4: 38 (25.3%),<br>Unknown: 12 (8%)
N-Stage	N0: 34 (22.7%),<br>N1: 23 (15.3%),<br>N2: 80 (53.3%),<br>N3: 2 (1.3%),<br>Unknown: 11 (7.3%)
M-Stage	M0: 149 (99.3%),<br>M1: 1 (0.7%)
HPV Status	Positive: 55 (36.7%),<br>Negative: 9 (6%),<br>Not recorded: 86 (57.3%)
Tumor Recurrence (Yes/No)	Yes: 46 (30.7%),<br>No: 104 (69.3%)

Abbreviations: ORN_0 is the date of the first clinical/radiologic detection of ORN. ORN_1 is the date at which the CT scan used in our study was taken. IMRT: Intensity-modulated radiotherapy. IMPT: Intensity-modulated proton therapy. VMAT: Volumetric modulated arc therapy. CT: Chemotherapy. CRT: Concurrent radiotherapy & chemotherapy.

* Heavy is defined as > 14 standard drinks per week for males or > 7 for females

** Last follow-up is considered the last clinical encounter with the patient in our institution.

Appendix B. Features selected through RFECV RF classifier.

Rank	Feature	Normal Mandible	ORN	P-Value	Importance
1	wavelet-LLH_firstorder_Median	-1.10 (-2.87, 0.00)	1.40 (0.52, 2.34)	< 0.01	0.062
2	wavelet-LLH_firstorder_Mean	-1.95 (-4.30, -0.79)	1.20 (-0.36, 3.05)	< 0.01	0.057
3	exponential_gldm_DependenceEntropy	6.87 (6.59, 7.12)	6.40 (6.15, 6.62)	< 0.01	0.049
4	wavelet-LLL_firstorder_90Percentile	817.43 (773.61, 856.19)	711.92 (659.39, 769.87)	< 0.01	0.044
5	square_firstorder_Kurtosis	2.03 (1.71, 2.75)	3.50 (2.55, 4.68)	< 0.01	0.041
6	square_firstorder_90Percentile	177.60 (161.03, 190.71)	135.85 (116.22, 157.83)	< 0.01	0.039
7	square_glrlm_LongRunHighGrayLevelEmphasis	1225.17 (919.73, 1539.25)	694.74 (538.29, 875.75)	< 0.01	0.035
8	wavelet-LLL_firstorder_InterquartileRange	311.33 (268.92, 351.93)	227.69 (181.54, 269.89)	< 0.01	0.032
9	exponential_firstorder_Skewness	0.98 (0.52, 1.49)	1.93 (1.41, 2.54)	< 0.01	0.029
10	exponential_firstorder_RootMeanSquared	24.87 (19.76, 29.91)	15.95 (13.31, 20.06)	< 0.01	0.028
11	exponential_ngtdm_Contrast	0.12 (0.07, 0.16)	0.04 (0.03, 0.08)	< 0.01	0.027
12	gradient_glcm_Idmn	0.96 (0.95, 0.97)	0.97 (0.97, 0.98)	< 0.01	0.026
13	exponential_glcm_Autocorrelation	57.78 (37.06, 81.57)	22.07 (16.02, 34.62)	< 0.01	0.025
14	exponential_glrlm_GrayLevelNonUniformityNormalized	0.09 (0.08, 0.11)	0.14 (0.12, 0.18)	< 0.01	0.025

15	square_firstorder_Skewness	0.44<br>(0.09, 0.86)	1.16<br>(0.80, 1.49)	< 0.01	0.023
16	exponential_firstorder_Kurtosis	2.80<br>(2.10, 4.74)	6.94<br>(4.13, 11.39)	< 0.01	0.021
17	gradient_ngtdm_Contrast	0.72<br>(0.50, 0.99)	0.47<br>(0.36, 0.57)	< 0.01	0.019
18	square_glrlm_RunEntropy	6.02<br>(5.79, 6.14)	5.70<br>(5.51, 5.85)	< 0.01	0.018
19	wavelet-LLH_firstorder_90Percentile	31.39<br>(27.55, 37.45)	38.47<br>(32.91, 44.74)	< 0.01	0.017
20	gradient_glcm_Contrast	263.84<br>(167.73, 334.23)	167.93<br>(115.00, 215.15)	< 0.01	0.016
21	square_firstorder_InterquartileRange	97.48<br>(76.20, 112.30)	55.39<br>(39.96, 75.47)	< 0.01	0.015
22	square_gldm_LargeDependenceHighGrayLevelEmphasis	6611.11<br>(4109.85, 10103.91)	3578.02<br>(2454.21, 4748.14)	< 0.01	0.014
23	gradient_firstorder_Kurtosis	2.76<br>(2.34, 3.51)	3.25<br>(2.70, 3.78)	< 0.01	0.014
24	exponential_glcm_JointEnergy	0.05<br>(0.02, 0.10)	0.14<br>(0.10, 0.21)	< 0.01	0.013
25	square_glcm_JointAverage	26.74<br>(22.33, 31.09)	18.22<br>(15.78, 21.78)	< 0.01	0.013
26	square_glcm_SumSquares	301.68<br>(242.97, 381.81)	183.56<br>(136.51, 250.74)	< 0.01	0.013
27	wavelet-HLL_glcm_MCC	0.58<br>(0.52, 0.65)	0.55<br>(0.50, 0.59)	< 0.01	0.013
28	gradient_glcm_DifferenceVariance	110.70<br>(78.09, 143.84)	74.90<br>(55.00, 100.18)	< 0.01	0.012
29	exponential_glrlm_LongRunHighGrayLevelEmphasis	112.34<br>(84.37, 148.09)	69.26<br>(53.87, 91.57)	< 0.01	0.011
30	wavelet-LLL_firstorder_Skewness	0.14<br>(-0.23, 0.46)	0.63<br>(0.35, 0.91)	< 0.01	0.011

31	wavelet-HLL_firstorder_Median	-0.75<br>(-2.00, 0.35)	0.09<br>(-0.55, 0.47)	< 0.01	0.010
32	square_firstorder_Median	73.87<br>(55.38, 103.63)	44.23<br>(35.48, 56.59)	< 0.01	0.010
33	wavelet-LLL_firstorder_Kurtosis	1.96<br>(1.77, 2.43)	2.69<br>(2.20, 3.39)	< 0.01	0.010
34	exponential_glcm_MaximumProbability	0.20<br>(0.11, 0.30)	0.35<br>(0.28, 0.43)	< 0.01	0.009
35	wavelet-LLH_firstorder_Range	225.40<br>(190.42, 269.12)	253.11<br>(215.54, 290.53)	< 0.01	0.009
36	squareroot_firstorder_InterquartileRange	94.05<br>(79.64, 107.28)	74.94<br>(59.08, 87.19)	< 0.01	0.009
37	wavelet-LLL_glszm_SizeZoneNonUniformityNormalized	0.72<br>(0.69, 0.75)	0.71<br>(0.68, 0.75)	0.715	0.009
38	wavelet-LLH_glszm_SizeZoneNonUniformityNormalized	0.48<br>(0.45, 0.51)	0.50<br>(0.46, 0.53)	< 0.01	0.009
39	exponential_gldm_DependenceVariance	37.96<br>(23.26, 52.92)	57.36<br>(45.18, 71.97)	< 0.01	0.008
40	wavelet-HLH_firstorder_Mean	0.04<br>(-0.01, 0.12)	0.00<br>(-0.07, 0.06)	< 0.01	0.008
41	logarithm_firstorder_InterquartileRange	48.06<br>(40.31, 57.05)	41.90<br>(33.90, 48.22)	< 0.01	0.008
42	logarithm_glcm_Imc1	-0.21<br>(-0.24, -0.19)	-0.19<br>(-0.22, -0.17)	< 0.01	0.008
43	squareroot_firstorder_Minimum	193.13<br>(160.02, 212.86)	119.76<br>(-54.24, 201.62)	< 0.01	0.008
44	wavelet-LLL_firstorder_Minimum	241.47<br>(185.86, 272.54)	134.06<br>(-5.65, 247.62)	< 0.01	0.008
45	logarithm_glszm_LargeAreaHighGrayLevelEmphasis	165209.55<br>(63935.56, 582780.65)	240066.10<br>(26617.54, 1869769.94)	0.973	0.007

46	wavelet-LLL_glcm_SumSquares	3053.63<br>(2535.92, 3761.31)	2336.29<br>(1711.19, 2976.32)	< 0.01	0.007
47	original_glszm_SizeZoneNonUniformityNormalized	0.55<br>(0.53, 0.58)	0.56<br>(0.53, 0.59)	0.612	0.007
48	square_ngtdm_Contrast	0.55<br>(0.41, 0.76)	0.30<br>(0.22, 0.47)	< 0.01	0.007
49	gradient_gldm_LargeDependenceHighGrayLevelEmphasis	2066.87<br>(1733.78, 2428.37)	1686.51<br>(1441.37, 1949.07)	< 0.01	0.006
50	square_glcm_JointEnergy	0.00<br>(0.00, 0.00)	0.01<br>(0.00, 0.01)	< 0.01	0.006
51	wavelet-LLH_firstorder_Maximum	105.05<br>(86.84, 130.16)	120.79<br>(98.98, 138.89)	< 0.01	0.006
52	wavelet-HHH_glszm_SizeZoneNonUniformityNormalized	0.45<br>(0.41, 0.47)	0.45<br>(0.42, 0.47)	0.927	0.006
53	square_glcm_Imc1	-0.21<br>(-0.25, -0.19)	-0.21<br>(-0.24, -0.17)	0.1	0.006
54	exponential_glcm_Imc2	0.86<br>(0.83, 0.89)	0.82<br>(0.77, 0.85)	< 0.01	0.006
55	wavelet-LLH_glszm_GrayLevelNonUniformityNormalized	0.03<br>(0.03, 0.03)	0.03<br>(0.02, 0.03)	0.012	0.005
56	wavelet-LHL_firstorder_Mean	-0.80<br>(-1.53, -0.39)	-0.73<br>(-1.27, -0.36)	0.27	0.005
57	gradient_glcm_ClusterShade	13095.96<br>(7712.06, 18856.09)	7795.60<br>(4734.61, 13476.16)	< 0.01	0.005
58	logarithm_glcm_MaximumProbability	0.03<br>(0.02, 0.04)	0.02<br>(0.01, 0.03)	0.015	0.005
59	wavelet-HHH_glcm_Imc1	-0.03<br>(-0.04, -0.02)	-0.02<br>(-0.03, -0.01)	< 0.01	0.005
60	wavelet-LLL_glcm_Idmn	0.98<br>(0.97, 0.99)	0.99<br>(0.98, 0.99)	< 0.01	0.004
61	gradient_glcm_ClusterProminence	1453769.21	846369.68	< 0.01	0.004

		(878408.89, 2255878.50)	(503966.68, 1431093.74)		
62	exponential_glcm_MCC	0.79<br><br>(0.74, 0.84)	0.77<br><br>(0.71, 0.81)	< 0.01	0.004
63	wavelet-LLH_glcm_DifferenceEntropy	4.24<br><br>(4.01, 4.43)	4.28<br><br>(4.11, 4.52)	0.075	0.004
64	logarithm_glcm_Idmn	0.98<br><br>(0.97, 0.99)	0.99<br><br>(0.98, 1.00)	< 0.01	0.004
65	square_glszm_GrayLevelNonUniformityNormalized	0.02<br><br>(0.02, 0.02)	0.02<br><br>(0.02, 0.03)	< 0.01	0.003
66	squareroot_ngtdm_Contrast	0.47<br><br>(0.33, 0.66)	0.32<br><br>(0.20, 0.51)	< 0.01	0.003
67	squareroot_glcm_Imc1	-0.21<br><br>(-0.25, -0.19)	-0.20<br><br>(-0.25, -0.17)	0.12	0.002

Appendix C. TRIPOD+AI checklist

TRIPOD+AI

Version: 11-January-2024

Section/Topic	Item	Development / evaluation[1]	Checklist item	Reported on page
**TITLE**				
Title	1	D;E	Identify the study as developing or evaluating the performance of a multivariable prediction model, the target population, and the outcome to be predicted	1
**ABSTRACT**				
Abstract	2	D;E	See TRIPOD+AI for Abstracts checklist	
**INTRODUCTION**				
Background	3a	D;E	Explain the healthcare context (including whether diagnostic or prognostic) and rationale for developing or evaluating the prediction model, including references to existing models	2
	3b	D;E	Describe the target population and the intended purpose of the prediction model in the context of the care pathway, including its intended users (e.g., healthcare professionals, patients, public)	2/3
	3c	D;E	Describe any known health inequalities between sociodemographic groups	
Objectives	4	D;E	Specify the study objectives, including whether the study describes the development or validation of a prediction model (or both)	3
**METHODS**				
Data	5a	D;E	Describe the sources of data separately for the development and evaluation datasets (e.g., randomised trial, cohort, routine care or registry data), the rationale for using these data, and representativeness of the data	3
	5b	D;E	Specify the dates of the collected participant data, including start and end of participant accrual; and, if applicable, end of follow-up	3
Participants	6a	D;E	Specify key elements of the study setting (e.g., primary care, secondary care, general population) including the number and location of centres	3
	6b	D;E	Describe the eligibility criteria for study participants	3/4
	6c	D;E	Give details of any treatments received, and how they were handled during model development or evaluation, if relevant	
Data preparation	7	D;E	Describe any data pre-processing and quality checking, including whether this was similar across relevant sociodemographic groups	4/5/6
Outcome	8a	D;E	Clearly define the outcome that is being predicted and the time horizon, including how and when assessed, the rationale for choosing this outcome, and whether the method of outcome assessment is consistent across sociodemographic groups	3
	8b	D;E	If outcome assessment requires subjective interpretation, describe the qualifications and demographic characteristics of the outcome assessors	
	8c	D;E	Report any actions to blind assessment of the outcome to be predicted	
Predictors	9a	D	Describe the choice of initial predictors (e.g., literature, previous models, all available predictors) and any pre-selection of predictors before model building	6
	9b	D;E	Clearly define all predictors, including how and when they were measured (and any actions to blind assessment of predictors for the outcome and other predictors)	6
	9c	D;E	If predictor measurement requires subjective interpretation, describe the qualifications and demographic characteristics of the predictor assessors	
Sample size	10	D;E	Explain how the study size was arrived at (separately for development and evaluation), and justify that the study size was sufficient to answer the research question. Include details of any sample size calculation	4/6
Missing data	11	D;E	Describe how missing data were handled. Provide reasons for omitting any data	
Analytical methods	12a	D	Describe how the data were used (e.g., for development and evaluation of model performance) in the analysis, including whether the data were partitioned, considering any sample size requirements	6/7
	12b	D	Depending on the type of model, describe how predictors were handled in the analyses (functional form, rescaling, transformation, or any standardisation).	6/7
	12c	D	Specify the type of model, rationale[2], all model-building steps, including any hyperparameter tuning, and method for internal validation	6/7
	12d	D;E	Describe if and how any heterogeneity in estimates of model parameter values and model performance was handled and quantified across clusters (e.g., hospitals, countries). See TRIPOD-Cluster for additional considerations[3]	
	12e	D;E	Specify all measures and plots used (and their rationale) to evaluate model performance (e.g., discrimination, calibration, clinical utility) and, if relevant, to compare multiple models	7
	12f	E	Describe any model updating (e.g., recalibration) arising from the model evaluation, either overall or for particular sociodemographic groups or settings	
	12g	E	For model evaluation, describe how the model predictions were calculated (e.g., formula, code, object, application programming interface)	7
Class imbalance	13	D;E	If class imbalance methods were used, state why and how this was done, and any subsequent methods to recalibrate the model or the model predictions	
Fairness	14	D;E	Describe any approaches that were used to address model fairness and their rationale	
Model output	15	D	Specify the output of the prediction model (e.g., probabilities, classification). Provide details and rationale for any classification and how the thresholds were identified	7-10

[1] D=items relevant only to the development of a prediction model; E=items relating solely to the evaluation of a prediction model; D;E=items applicable to both the development and evaluation of a prediction model
[2] Separately for all model building approaches.
[3] TRIPOD-Cluster is a checklist of reporting recommendations for studies developing or validating models that explicitly account for clustering or explore heterogeneity in model performance (eg, at different hospitals or centres). Debray et al, BMJ 2023; 380: e071018 [DOI: 10.1136/bmj-2022-071018]

Training versus evaluation	16	D;E	Identify any differences between the development and evaluation data in healthcare setting, eligibility criteria, outcome, and predictors	
Ethical approval	17	D;E	Name the institutional research board or ethics committee that approved the study and describe the participant-informed consent or the ethics committee waiver of informed consent	3
**OPEN SCIENCE**				
Funding	18a	D;E	Give the source of funding and the role of the funders for the present study	12/13
Conflicts of interest	18b	D;E	Declare any conflicts of interest and financial disclosures for all authors	13
Protocol	18c	D;E	Indicate where the study protocol can be accessed or state that a protocol was not prepared	
Registration	18d	D;E	Provide registration information for the study, including register name and registration number, or state that the study was not registered	
Data sharing	18e	D;E	Provide details of the availability of the study data	13
Code sharing	18f	D;E	Provide details of the availability of the analytical code[4]	
**PATIENT & PUBLIC INVOLVEMENT**				
Patient & Public Involvement	19	D;E	Provide details of any patient and public involvement during the design, conduct, reporting, interpretation, or dissemination of the study or state no involvement.	
**RESULTS**				
Participants	20a	D;E	Describe the flow of participants through the study, including the number of participants with and without the outcome and, if applicable, a summary of the follow-up time. A diagram may be helpful.	4
	20b	D;E	Report the characteristics overall and, where applicable, for each data source or setting, including the key dates, key predictors (including demographics), treatments received, sample size, number of outcome events, follow-up time, and amount of missing data. A table may be helpful. Report any differences across key demographic groups.	7
	20c	E	For model evaluation, show a comparison with the development data of the distribution of important predictors (demographics, predictors, and outcome).	
Model development	21	D;E	Specify the number of participants and outcome events in each analysis (e.g., for model development, hyperparameter tuning, model evaluation)	4
Model specification	22	D	Provide details of the full prediction model (e.g., formula, code, object, application programming interface) to allow predictions in new individuals and to enable third-party evaluation and implementation, including any restrictions to access or re-use (e.g., freely available, proprietary)[5]	6
Model performance	23a	D;E	Report model performance estimates with confidence intervals, including for any key subgroups (e.g., sociodemographic). Consider plots to aid presentation.	8/9
	23b	D;E	If examined, report results of any heterogeneity in model performance across clusters. See TRIPOD Cluster for additional details[3].	
Model updating	24	E	Report the results from any model updating, including the updated model and subsequent performance	
**DISCUSSION**				
Interpretation	25	D;E	Give an overall interpretation of the main results, including issues of fairness in the context of the objectives and previous studies	11/12
Limitations	26	D;E	Discuss any limitations of the study (such as a non-representative sample, sample size, overfitting, missing data) and their effects on any biases, statistical uncertainty, and generalizability	11/12
Usability of the model in the context of current care	27a	D	Describe how poor quality or unavailable input data (e.g., predictor values) should be assessed and handled when implementing the prediction model	11/12
	27b	D	Specify whether users will be required to interact in the handling of the input data or use of the model, and what level of expertise is required of users	
	27c	D;E	Discuss any next steps for future research, with a specific view to applicability and generalizability of the model	11/12

From: Collins GS, Moons KGM, Dhiman P, et al. *BMJ* 2024;385:e078378. doi:10.1136/bmj-2023-078378

[4] This relates to the analysis code, for example, any data cleaning, feature engineering, model building, evaluation.
[5] This relates to the code to implement the model to get estimates of risk for a new individual.
